# Refining APP strategies in COVID-19: balancing efficacy, feasibility, and individual needs

**DOI:** 10.1186/s13613-025-01540-1

**Published:** 2025-08-11

**Authors:** Qin Sun, Hui Chen, Yi Yang, Haibo Qiu, Ling Liu

**Affiliations:** Jiangsu Provincial Key Laboratory of Critical Care Medicine, Department of Critical Care Medicine, School of Medicine, Zhongda Hospital, Southeast University, 87 Dingjiaqiao Rd., Nanjing, 210009 China

We sincerely thank the authors for their constructive comments on our recent secondary analysis investigating the association between the duration of awake prone positioning (APP) and clinical outcomes in patients with COVID-19-related acute hypoxemic respiratory failure (AHRF) [1, 2]. We appreciate the perspectives raised in the commentary and address them point by point below. Their letter raises important considerations that merit further discussion.

We fully acknowledge that viral variants and vaccination status can impact the clinical course of COVID-19, and our study was conducted primarily during the circulation of the SARS-CoV-2 Omicron variant. However, our primary objective was to assess the impact of APP duration on clinical outcomes. Previous studies conducted during periods dominated by variants such as Alpha and Delta have not demonstrated that different variants modify the effect of APP on intubation rates [3]. Therefore, we do not believe the circulating variant significantly influenced our study conclusions.

Considering vaccination status does not directly affect the actual duration of APP in our cohort, it was not included as a confounding factor in the main analysis. Nevertheless, in response to concerns raised by Yisheng Cao and colleague [1], we reanalyzed the data. The vaccination rates in the APP success and failure groups were 81.5% and 69.5%, respectively (247/303 vs. 73/105, *P* = 0.015). After incorporating vaccination status into multivariate models and E-value sensitivity analyses, our primary results remained robust.

Different non-invasive respiratory support strategies and their potential effects may indeed influence APP duration. In our primary analysis, non-invasive respiratory support (including HFNC and NIV) was an independent risk factor for APP failure (HR 6.04, 95% CI 1.68–21.78). As noted in the manuscript, this variable was adjusted for in the multivariable Cox model, and the interaction between NIV and APP duration was examined in Supplementary Tables S2–S4 [2]. We have now further analyzed the interaction between HFNC and APP duration and found no significant interactions for either HFNC or NIV. The only significant interaction observed was between treatment allocation group and APP duration. Of course, these findings are limited by the secondary nature of the analysis. Future studies should further investigate how different respiratory support strategies may modulate the efficacy of APP and help identify subgroups most likely to benefit from individualized approaches.

Maintaining 8–12 h of daily APP can be challenging, particularly in elderly, frail patients or those with comorbidities such as chronic pain or anxiety. However, Matías and colleagues showed that implementing a care bundle—including light sedation, monitoring, and patient education—can significantly increase APP duration [4]. Our previous RCT also outlined strategies to improve adherence and extend APP time (see Table 1) [5]. Furthermore, an individual participant data meta-analysis demonstrated that with adequate supportive measures, patients can achieve longer APP durations, and maintaining ≥ 10 h/day was associated with improved outcomes [3]. Future work should explore alternative positioning strategies (e.g., lateral or intermittent APP) and interventions aimed at enhancing patient comfort to support broader application of APP.

In conclusion, we thank the authors again for their valuable insights, which contribute meaningfully to advancing the discussion from identifying the “optimal protocol” to determining the “most appropriate population” for APP. We believe this dialogue will help refine and personalize this important intervention.
